# A 5-year trend in the use of sodium-glucose co-transporter 2 inhibitors and other oral antidiabetic drugs in a Middle Eastern country

**DOI:** 10.1007/s11096-022-01464-x

**Published:** 2022-09-28

**Authors:** Nancy Zaghloul, Ahmed Awaisu, Ahmed Mahfouz, Sumaya Alyafei, Hazem Elewa

**Affiliations:** 1 Pharmacy Department, Heart Hospital, Hamad Medical Corporation, 3050 Doha, Qatar; 2 Biomedical and Pharmaceutical Research Unit, Clinical Pharmacy and Practice Department, College of Pharmacy, QU Health, Qatar University, 2713 Doha, Qatar; 3 College of Pharmacy and Health Sciences, Butler University, Indianopolis, USA

**Keywords:** Diabetes mellitus, Hypoglycemic agents, Qatar, Sodium-glucose transporter 2 inhibitors, Trends, Type 2

## Abstract

**Background:**

Sodium glucose co-transporter 2 inhibitors (SGLT2is) are a novel class of oral antidiabetic drugs. To date, there are no pharmacoepidemiologic studies investigating the pattern of use of SGLT2is compared to other oral antidiabetic drugs in the Middle East, including Qatar.

**Aim:**

This study aimed to explore the trends in the use of SGLT2is compared to other oral antidiabetic drugs in Qatar from 2016 to 2020.

**Method:**

This is a descriptive, retrospective cross-sectional study where information on all oral antidiabetic drugs dispensed as in- or out-patient prescriptions from 2016 to 2020 in Hamad Medical Corporation hospitals, Qatar were collected. Outcomes included the number and relative frequency of quarterly prescriptions of different oral antidiabetic drug classes [biguanides, sulfonylureas, dipeptidyl peptidase 4 inhibitors, thiazolidinediones, meglitinides, α-glucosidase inhibitors, and SGLT2is] prescribed from 2016 to 2020.

**Results:**

SGLT2is prescriptions increased from 1045 (2.13%) in 2017 to 8375 (12.39%) in 2020, while sulfonylureas prescriptions declined from 10,436 (21.25%) to 9158 (13.55%) during the same period. Metformin use decreased from 23,926 (48.71%) in 2017 to 30,886 (45.70%) in 2020. The proportions of thiazolidinediones, meglitinides, α-glucosidase inhibitors prescriptions remained stable over the years. Among SGLT2is, empagliflozin prescriptions showed an increase from 537 (10.65%) to 2881 (34.40%) compared to dapagliflozin, which decreased by the end of 2018 from 4505 (89.35%) to 5494 (65.6%).

**Conclusion:**

SGLT2is have largely replaced sulfonylureas in Qatar. The increasing trend in their use over the years is similar to that reported in other countries. The trend among SGLT2is suggests greater preference for empagliflozin over dapagliflozin.

## Impact statements

- In Qatar, the use of sodium-glucose cotransporter 2 inhibitors is on the rise given their proven benefits.
- Clinicians should be up-to-date on how to effectively prescribe these medications and be knowledgeable with their place in therapy.
- Findings from this study can help healthcare policy makers to recognize the trends in the utilization of new antidiabetic drug classes added to the formulary in order to determine the need to add more agents from various classes.

## Introduction

Over the past few decades, the unmet needs in diabetes management have led to the discovery of new therapeutic targets and drug classes, including sodium-glucose co-transporter 2 inhibitors (SGLT2is), dipeptidyl peptidase 4 inhibitors (DPP4is), incretin-based therapies, and insulin analogues. Consequently, the introduction of these new agents has resulted in a marked change in the prescribing of antidiabetic drugs (ADDs) for patients with type 2 diabetes mellitus (T2DM) in recent years. Similarly, evidence-based clinical practice guidelines for the management of diabetes have undergone significant updates to include greater options of ADDs when glycemic control is not achieved with metformin monotherapy [1–5].

Diabetes mellitus (DM) is a major public health issue that affects more than 400 million adults worldwide [6]. Diabetes can be classified into type 1 diabetes mellitus (T1DM), T2DM, and gestational diabetes [6]. T1DM affects about 10% of the diabetic population [7], while T2DM accounts for about 90% of all cases of diabetes [8]. Around 14% of pregnant women have gestational diabetes [9]. In 2021, the global diabetes prevalence was estimated to be 10.5% (536.6 million people), and it is projected to increase to 12.2% (783.2 million) in 2045 [10]. 6.7 million death cases were attributed to diabetes in 2021 [11]. Global health expenditures related to diabetes were approximated at 966 billion USD in 2021, and are expected to reach 1054 billion USD in 2045 [10]. It was reported that 73 million adults live with diabetes in the Middle-East and North Africa in 2021, and this number is anticipated to increase to 136 million by 2045 [11]. Equally, diabetes represents a growing public health challenge in Qatar. For instance, in 2020, the prevalence of DM among Qatari population was reported as 15.5% [12]. It is apparent that, if concerted efforts and necessary actions are not undertaken, this prevalence is anticipated to increase by more than double in the 35–60 age group by 2045 [13]. DM is associated with a high risk for cardiovascular (CV) morbidity and mortality [14, 15] and progression of renal disease [14]. Despite advances in secondary prevention therapy for cardiovascular diseases (CVDs), patients with diabetes are still at risk of complications from atherosclerotic cardiovascular disease (ASCVD) and kidney disease [14, 15]. Therefore, the major goals of therapy for diabetes are to prevent or delay microvascular and macrovascular complications and to improve quality of life [16].

SGLT2is are a novel class of oral ADDs. They mediate their effect by blocking glucose renal reabsorption, thus promoting urinary glucose excretion and reducing blood glucose levels [17]. The increase in glucosuria also provides several metabolic benefits, including, reduction of glycosylated hemoglobin (HbA_1c_), blood pressure, body weight, and albuminuria [18]. On the other hand, SGLT2is have been associated with an increase in low-density lipoprotein (LDL) and high-density lipoprotein (HDL) cholesterols [19], and genitourinary infections [20]. They may also increase the risk of amputations [21, 22], metabolic acidosis [23, 24], and bone fractures [25]. In addition, they are contraindicated in patients with severe renal impairment, end-stage renal disease, or in those receiving dialysis [26–28]. Canagliflozin was the first agent to be approved for the treatment of T2DM by the United States Food and Drug Administration (U.S. FDA) and the European Medicines Agency (EMA) in 2013 [26, 29]. This was followed by the approval of dapagliflozin and empagliflozin in 2014 [27, 28, 30, 31]. Ertugliflozin was the last among these agents to be approved in 2018 [32, 33]. Clinical practice guidelines have recommended SGLT2is as an add-on or second-line therapy after metformin monotherapy failure or intolerance [1–5], especially in patients with established ASCVD [2, 4, 34], heart failure with reduced ejection fraction [35], and chronic kidney disease [36, 37].

While emerging evidence and treatment guidelines have the largest influence on prescribing patterns, trends in real-world may be influenced by other factors, such as clinician’s familiarity, medication costs, patient preferences, and adverse effects concerns [38–40]. These patterns and trends in the utilization of ADDs could vary from one region or country to another, depending on several factors. Recent pharmacoepidemiological studies from the U.S., Europe, and East Asia have demonstrated changes in prescribed ADDs as add-on to metformin, where there was a decline in the use of sulfonylureas (SUs), an increase in the use of dipeptidyl peptidase 4 inhibitors (DPP4is), and a slight increase in SGLT2is [41–47]. To our knowledge, there are no pharmacoepidemiologic studies investigating the pattern of use of SGLT2is in the region of the Middle East, including Qatar. The Middle East may differ from the Western countries in terms of the clinical settings and the local clinical practice guidelines. Moreover, there is a diversity in the nationalities of healthcare providers in the Middle East, and particularly in the Gulf region, where clinicians come from different medical backgrounds with various experiences [48]. These differences could contribute to different prescribing patterns. Additionally, the majority of the published studies from elsewhere were conducted before expanding the drug label indication for dapagliflozin to include reducing the risk of CV death and hospitalization for HF in patients with reduced ejection fraction HF [49].

### Aim

This study aimed to explore the trends in the use of SGLT2is compared to other oral ADDs in Qatar over 5 years (i.e. from 2016 to 2020).

### Ethics approval

The Institutional Review Board (IRB) at Hamad Medical Corporation (HMC) (MRC-01–20-1055) and Qatar University (1471-E/21) approved the study protocol.

## Method

### Study design and setting

This is a descriptive, retrospective cross-sectional study on the 5-year utilization of the approved SGLT2is in HMC’s formulary (dapagliflozin 10 mg, and empagliflozin 10 mg and 25 mg) compared to other oral ADDs. HMC is the principal public healthcare provider in the State of Qatar and consists of 12 general and specialized hospitals. HMC facilities deliver medical services to more than 80% of the population in the State of Qatar.

### Data collection and study population

At HMC, dapagliflozin was added to the formulary in 2017, followed by empagliflozin in 2018. In this study, we collected information from electronic medical records on all oral ADDs [biguanides (BGs), sulfonylureas (SUs), dipeptidyl peptidase 4 inhibitors (DPP4is), thiazolidinediones (TZDs), meglitinides (MEGs), α-glucosidase inhibitors (AGIs), and sodium-glucose co-transporter 2 inhibitors (SGLT2is)] dispensed as in- or out-patient prescriptions from 1 January 2016 to 31 December 2020. Information on the usage of the oral ADDs in 2016 served as a baseline of their trend of use before HMC formulary’s inclusion of SGLT2is in 2017. Therefore, data from the beginning of 2017 to the end of 2020 included the usage of all oral ADDs, including the SGLT2is. The following data were extracted from the electronic medical record system (Cerner®) at HMC: the names of SGLT2is and other oral ADDs, patients’ demographics and baseline characteristics including age, gender, and nationality. Cerner® is considered a potential important resource for health research [50]. It is easy to access, provides comprehensive and clear data, and allows future follow up of long-term data. The data for each class of oral ADDs were extracted from Cerner® into Excel sheets. The extraction is considered to be very accurate and reflects the actual data since Cerner is the only system used to dispense medications at HMC. Additionally, “medications” and the other variables that were extracted (age, gender, nationalities) are structured data which reduces the opportunities for any false positive and/or false negative during the extraction process.

Then, prescriptions were stratified by quarter and the specific ADD class prescribed. Duplicates were removed from each quarter based on the patient’s medical record number to get the total number of prescriptions in each quarter. Data were screened for any missing information in each quarter. The overall number of study subjects was calculated by removing all the duplicates of patients who were using more than one class at a time.

Inclusion criteria included any oral ADDs prescriptions for adult patients (≥ 18 years old), between 2016 and 2020 at HMC. Prescriptions for patients < 18 years old, those for duration of less than 3 days, and those for insulin or glucagon-like peptide-1 receptor agonists were excluded. Insulin was excluded since it focuses more on T1DM population, while glucagon-like peptide-1 receptor agonists were excluded since their use is restricted to Qatari patients and are non-formulary for the majority of the patient population.

### Outcome measures

Outcomes were the number and relative frequency of quarterly prescriptions (every 3 months starting from first of January of each year) of different oral ADDs classes from 2016 to 2020. Quarterly prescriptions of SGLT2is (dapagliflozin and empagliflozin) users per quarter from 2017 to 2020 were also collected. In addition, the pattern of the different ADDs combinations and their frequencies were captured. The ADDs combinations were categorized into monotherapy, dual therapy, and triple therapy or more.

### Statistical analysis

Statistical Package for the Social Sciences (SPSS®) Version 27 (IBM SPSS®23 software) was used for descriptive statistical analyses of the collected data. Continuous variables were expressed as mean ± SD, while categorical variables were expressed as frequencies and percentages.

## Results

### Characteristics of the study patients

From 2016 to 2020, there were a total of 114,919 patients prescribed oral ADDs. Overall, more than half of the study subjects (57.5%) were less than 55 years old, and about 58% of them were male. The proportions of Arabs vs. non-Arabs were approximately equal at 56,905 (49.5%) and 58,014 (50.5%), respectively. The majority of the patients were on BGs (metformin) (n = 108,808;94.7%), followed by DPP4is (n = 48,611; 42.3%), SUs (n = 35,341; 30.8%), SGLT2is (n = 17,942; 15.6%), TZDs (7257; n = 6.3%), MEGS (n = 1523; 1.3%), and AGIs (n = 398; 0.3%) at any time between 2016 to 2020 (Table 1).

**Table 1:** Demographics and baseline characteristics of patients on oral antidiabetic drugs from 2016 to 2020

	BGs	SUs	DPP4is	TZDs	MEGs	AGIs	SGLT2is
Number of patients n (%)	108,808 (94.7)	35,341 (30.8)	48,611 (42.3)	7257 (6.3)	1523 (1.3)	398 (0.3)	17,942 (15.6)
Age (± SD) < 55 55–75 > 75	52 (± 15) 64,038 (58.9) 39,865 (36.6) 4905 (4.5)	56 (± 12) 15,722 (44.5) 17,488 (49.5) 2131 (6.0)	56 (± 12) 22,668 (46.6) 23,164 (47.7) 2779 (5.7)	56 (± 12) 3116 (42.9) 3815 (52.6) 326 (4.5)	63 (± 13) 348 (22.8) 921 (60.5) 254 (16.7)	57 (± 15) 166 (41.7) 200 (50.3) 32 (8.0)	55 (± 11) 8383 (46.7) 9005 (50.2) 554 (3.1)
Gender n (%)							
Male Female	62,439 (57.4) 46,369 (42.6)	25,062 (70.9) 10,279 (29.1)	33,376 (68.7) 15,235 (31.3)	4914 (67.7) 2343 (32.3)	883 (58.0) 640 (42.0)	200 (50.3) 198 (49.7)	11,320 (63.1) 6622 (36.9)
Nationality n (%)							
Arabs Non-Arabs	53,484 (49.2) 55,324 (50.8)	16,117 (45.6) 19,224 (54.4)	23,802 (49.0) 24,809 (51.0)	4000 (55.1) 3257 (44.9)	1114 (73.1) 409 (26.9)	274 (68.8) 124 (31.2)	10,698 (59.6) 7244 (40.4)

*SD*, standard deviation; *BG*s, biguanides (metformin); *SU*s, sulfonylureas; *DPP*4is, dipeptidyl peptidase 4 inhibitors; *TZD*s, thiazolidinediones; *MEG*s, meglitinides; *AGI*s, α-glucosidase inhibitors; *SGLT*2is, sodium-glucose co-transporter 2 inhibitors

The overall number of patients on antidiabetic drugs does not represent the addition of the number of patients on different classes of oral antidiabetic drugs since many of these patients were using more than one class at the same time

### Trends in the use of oral antidiabetic drugs

**Fig. 1:**
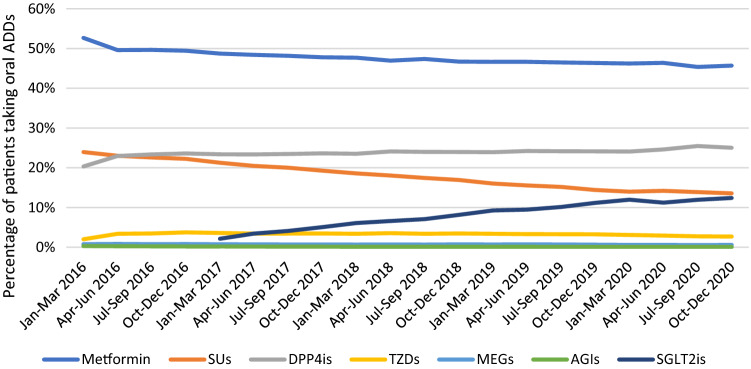
Trends in the use of oral antidiabetic drugs from 2016 to 2020. ADDs, antidiabetic drugs; BGs, biguanides (metformin); SUs, sulfonylureas; DPP4is, dipeptidyl peptidase 4 inhibitors; TZDs, thiazolidinediones; MEGs, meglitinides; AGIs, α-glucosidase inhibitors; SGLT2is, sodium-glucose co-transporter 2 inhibitors

**Fig. 2:**
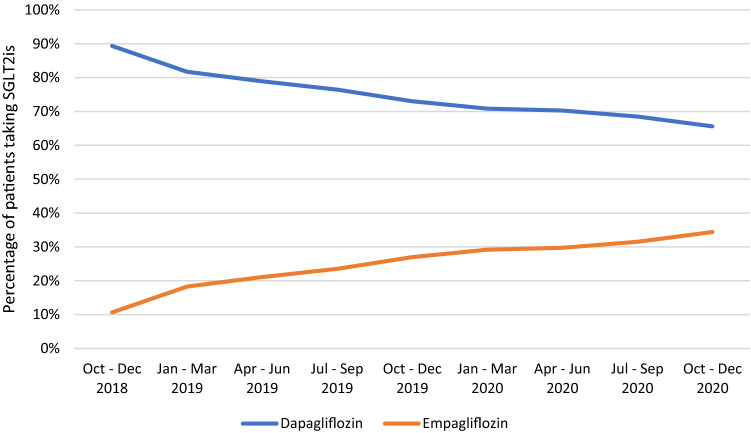
Trends in the use of sodium–glucose co-transporter 2 inhibitors from 2018 to 2020

The volume of oral ADDs prescriptions increased from 37,839 in 2016 to 62,797 in 2020. In 2016, the use of metformin was stable, while there was an increase in DPP4is use and a decrease in SUs use (Fig. 1). After being introduced to the formulary in 2017, SGLT2is prescriptions increased over the years from 1045 (2.13%) in 2017 to 8375 (12.39%) in 2020, while SUs prescriptions showed a decline from 10,436 (21.25%) to 9158 (13.55%) during the same period. In addition, metformin use decreased from 23,926 (48.71%) in 2017 to 30,886 (45.70%) in 2020. On the other hand, the proportions of TZDs, MEGs, and AGIs prescriptions remained stable over the years as shown in Fig. 1. Among SGLT2is, empagliflozin prescriptions showed an increase from 537 (10.65%) to 2881 (34.40%) compared to dapagliflozin, which decreased by the end of 2018 from 4505 (89.35%) to 5494 (65.6%) (Fig. 2).

### Trends in the use of oral antidiabetic drugs combination therapy

**Fig. 3:**
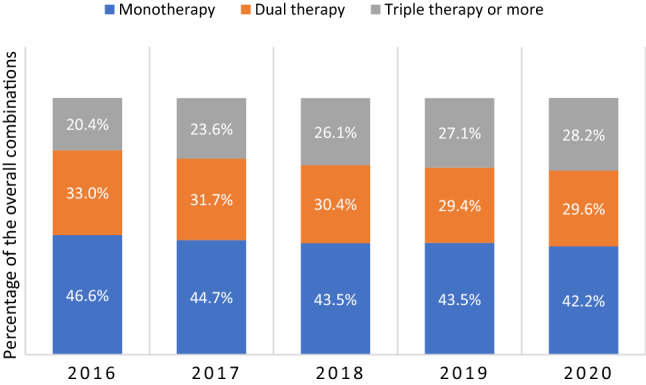
Trends of most frequently prescribed oral antidiabetic drugs combinations from 2016 to 2020

**Fig. 4:**
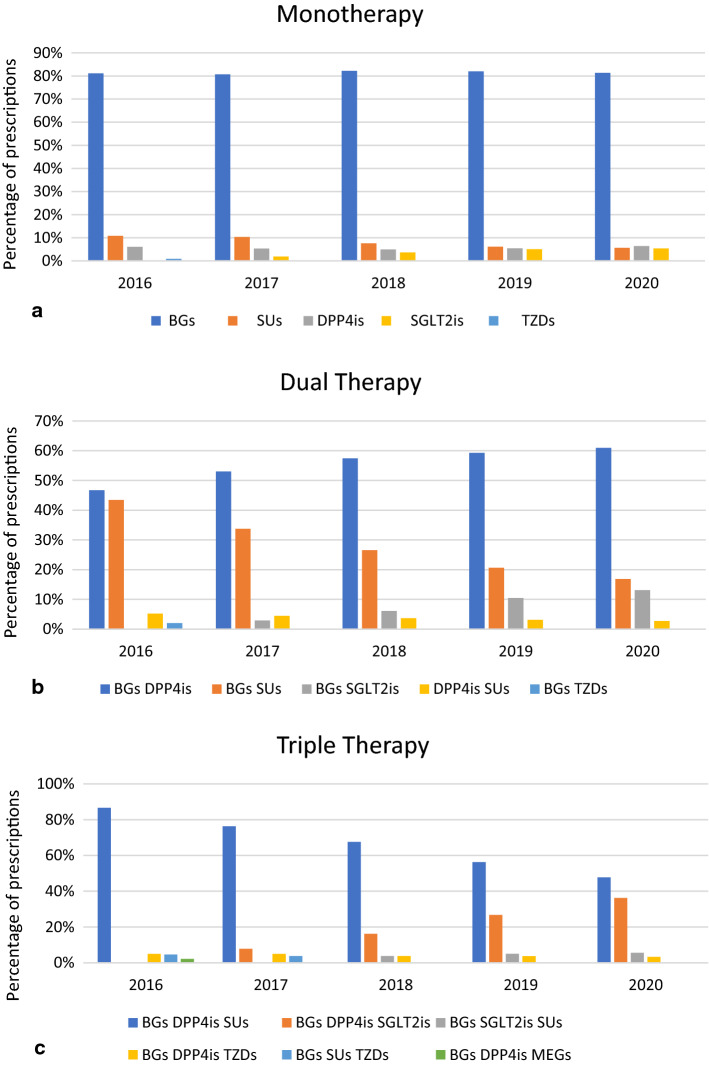
Trends of most frequently prescribed oral antidiabetic drugs combinations of **a** monotherapy, **b** dual therapy, and **c** triple therapy from 2016 to 2020. BGs, biguanides (metformin); SUs, sulfonylureas; DPP4is, dipeptidyl peptidase 4 inhibitors; TZDs, thiazolidinediones; MEGs, meglitinides; SGLT2is, sodium-glucose co-transporter 2 inhibitors

Figure 3 illustrates the utilization of combinations of oral ADDs among patients with T2DM in Qatar. Monotherapy decreased slightly from 46.62% in 2016 to 42.18% in 2020. Among patients receiving monotherapy, the most frequently prescribed ADD was metformin (81.12% in 2016 vs. 81.36% in 2020) (Fig. 4a). The subsequent most frequent monotherapies in 2016 were SUs, DPP4is, and TZDs. From 2017 to 2020, these were changed to SUs, DPP4is, and SGLT2is.

Similarly, the use of dual therapy decreased slightly from 32.99% in 2016 to 29.59% in 2020. The most frequent combinations among patients who received dual therapy in 2016 were BGs–DPP4is (46.73%), and BGs–SUs (43.45%). Since 2017 to 2020, BGs–SUs trended down consistently at the expense of an increase in BGs–SGLT2is, and BGs–DPP4is (Fig. 4b).

In contrast, the utilization of triple or more ADD combination therapy increased from 20.40% in 2016 to 28.24% in 2020. The most frequently used combination among patients who received triple therapy in 2016 was BGs–DPP4is–SUs (86.63%), which trended down to reach 47.75% in 2020. On the other hand, BGs–DPP4is–SGLT2is use increased dramatically from 7.84% in 2017 to 36.20% in 2020 (Fig. 4c).

## Discussion

### Statement of key findings

Our findings indicate that the use of oral ADDs significantly increased in Qatar over 5 years, which may be attributed to the increasing prevalence of DM in Qatar [13]. Following the addition of SGLT2is to HMC’s formulary in 2017, there was an increase in their uptake and a corresponding reduction primarily in the SUs. In addition, the utilization of SGLT2is showed an increase as monotherapy, dual therapy, and in triple or more ADD combination therapy.

### Interpretation

Metformin use declined from 48.71% in 2017 to 45.70% in 2020, which is in contrast to recent studies that showed either a stable or an increased trend in metformin use in other countries [41, 43, 46, 51]. Moreover, SUs prescribing considerably decreased from 2017 to 2020, and this coincided with a slight increase in DPP4is prescribing. The decrease in SUs prescribing is most likely explained by physicians' attempts to reduce their risk of hypoglycemia and weight gain [1], and the availability of safer ADDs. On the other hand, the increase in DPP4is use is expected as they do not cause hypoglycemia and weight gain [1]. The trends of SUs and DPP-4is prescribing and utilization are consistent with studies in other countries [41–44, 46, 52].

In line with the previous studies conducted elsewhere, a rapid increase in SGLT2is use was observed since they became available in HMC’s formulary in 2017 [41, 44, 46, 51, 52]. The main reason for the increased trend in the utilization of this novel class of ADDs is that they are associated with reductions in blood pressure, body weight, and albuminuria, and are not associated with hypoglycemia [1, 18]. Besides that, the emergence of evidence of CV and renal benefits through landmark cardiovascular outcomes trials [21, 53–58], which consequently led to expansion in their drug label by regulatory authorities [59, 60], could also be a potential contributing factor in the increased use of SGLT2is. The findings of EMPA-REG OUTCOME trial resulted in expanding the drug label for empagliflozin in December 2016 by the U.S. FDA to include reducing the risk of CV death in adult patients with T2DM and CVD [53, 59]. In addition, after publishing the results of DAPA-HF trial, the U.S. FDA approved dapagliflozin for reducing the risk of CV death and hospitalization for HF in patients with reduced ejection fraction in May 2020 [55, 60]. All of these advantages of SGLT2is clearly explain the reasons for their increased prescribing patterns over the years.

Among SGLT2is, we noted a significant increase in empagliflozin use over dapagliflozin before 2020. Although there are no head-to-head clinical trials comparing SGLT2is, a potential reason for the preference of empagliflozin over dapagliflozin could be due to the new indication of the former in 2016 to reduce the risk of CV death in adult patients with T2DM and CVD [59]. After that, the approximately parallel trends of both agents by the end of 2019 until the end of 2020 could be attributed to the published results of DAPA-HF trial in November 2019 that led to a new indication of dapagliflozin in 2020 to reduce the risk of CV death and hospitalization for HF in patients with reduced ejection fraction HF [60]. Similar results were shown by a study in a U.S. academic medical center [61].

Furthermore, the rate of monotherapy prescriptions decreased over time, which is in alignment with the rates reported in other countries [46, 52]. Likewise, the use of dual therapy decreased during the same period. In contrast, the prescription rates of combinations of 3 or more ADDs increased over time and this increase, which explains the decline in the rates of monotherapy and dual therapy, is consistent with a study conducted in Korea [52].

Metformin monotherapy or combination therapy was the most commonly prescribed medication over the five-year period of the study. This pattern is consistent with diabetes management guidelines, as metformin is considered the first line treatment for T2DM [1–5]. This finding is also consistent with other studies from the U.S., United Kingdom, and East Asia [41, 43, 46, 51, 52, 62]. Furthermore, a Korean study showed that the most common dual therapy was BGs–DPP4is, which is similar to our results [52]. Prior to introducing SGLT2is to HMC’s formulary, BGs–SUs combination was the second most frequently prescribed dual therapy. However, in 2017 there was a shift from BGs–SUs to BGs–SGLT2is, which continued to rise until 2020. Among triple therapy, BGs–DPP4is–SUs was the most commonly prescribed combination in 2016; however, this declined over the years following the introduction of SGLT2is. This decline was offset by a gradual increase in BGs–DPP4is–SGLT2is. Given the advantages and the definitive evidence of benefit of SGLT2is, the shift from SUs–based to SGLT2is–based combinations was expected.

### Strengths and weaknesses

A major strength in our study is that it provides a detailed explanation on the trend in the use of various oral ADDs and the pattern of various ADDs combinations in Qatar. To the best of our knowledge, this is the only study with such focus in Qatar and the Middle East especially after the introduction of SGLT2is. However, our study is not without limitations. The most important limitation is that we did not include data from community pharmacies and private hospitals. Therefore, caution should be exercised when generalizing these findings. Additionally, the gathered demographics data of the study did not did include descriptors other than the age, gender and nationality. Furthermore, the paper lacks inferential statistical analyses as there are no independent groups with different exposures to compare for a certain outcome or an association that we aimed to test for.

### Further research

Future studies can focus on investigating the factors associated with prescribing SGLT2is compared to other ADDs, as well as the factors associated with prescribing a specific agent under this class.

## Conclusion

SGLT2is have shown an increasing prescribing trend in Qatar over the years which is similar to that reported in other countries. The trend among SGLT2is suggests greater preference for empagliflozin over dapagliflozin. Meanwhile, the use of SUs declined over time. Additionally, SGLT2is prescriptions showed an increase as monotherapy, dual therapy, and in triple or more ADD combination therapy.
